# Reducing Antibiotic Use for Young Children with Intussusception following Successful Air Enema Reduction

**DOI:** 10.1371/journal.pone.0142999

**Published:** 2015-11-16

**Authors:** Yuan Zhang, Wen Zou, Yinghui Zhang, Weimin Ye, Xingdong Chen, Qian Liu, Huandi Liu, Chunfeng Si, Hongying Jia

**Affiliations:** 1 Center of Evidence-based Medicine, Second Hospital of Shandong University, Jinan, Shandong, China; 2 Department of Pharmacy, Second Hospital of Shandong University, Jinan, Shandong, China; 3 Department of Medical Records, Second Hospital of Shandong University, Jinan, Shandong, China; 4 Department of Medical Epidemiology and Biostatistics, Karolinska Institutet, Solna, Stockholm, Sweden; 5 Department of Pediatric Surgery, Second Hospital of Shandong University, Jinan, Shandong, China; 6 Information Center, Second Hospital of Shandong University, Jinan, Shandong, China; Nottingham University, UNITED KINGDOM

## Abstract

China introduced a new policy regarding the management of antibiotic use. We evaluated the reasonableness of antibiotic use among children suffering from intussusception before and after policy. A retrospective study was conducted involving 234 young children with intussusception who were treated between January 1, 2011 and December 30, 2013. Demographics and detailed antibiotics regimens were collected. *χ* ^*2*^ test was used to evaluate differences between the phase I (preintervention, n = 68) and phase II (postintervention, n = 166). We determined that the overall antibiotic use rate following successful air enema reduction was 41% (97/234), which decreased from 99% (67/68) in phase I to 18% (30/166) in phase II. In phase I, prophylactic antibiotic usage reached up to 84% (56/67). The quantity of aztreonam for injection accounted for 63% (45/71), and cefamandole nafate for injection accounted for 25% (18/71). In phases II, prophylactic antibiotic usage were reduced to 13% (4/30). The quantity of aztreonam for injection was decreased to 12% (4/33) and cefamandole nafate for injection was 3% (1/33). Antibiotics' options were more diverse. In conclusion, policy intervention was effective in addressing some aspects of antibacterial drug usage among young children with intussusception. However, excessive drug use remains a public health problem. The guidelines for the antibiotic management of intussusception for children must be established in China.

## Introduction

Intussusception occurs worldwide; its incidence is approximately 1 to 4 out of every 2000 infants, and with a peak prevalence within their first 3 years of life [1]. It is a pediatric emergency and the second most common cause of gastrointestinal obstruction among young children. It occurs when a segment of bowel (the intussusceptum) telescopes into a more distal bowel segment (the intussuscipiens), resulting in both venous congestion and bowel wall edema [2]. As a non-operative technique, air enema reduction is quick and clean, and is associated with a high reduction rate (73%–95%) [3]. However, the management of antibiotics for intussusception remains variable. Whether antibiotics should be applied prophylactically, either before or after reduction, to prevent infectious complications remains controversial worldwide [1,4,5]. In China, there were no evidence-based guidelines regarding antibiotic usage in the setting of intussusception. Moreover, the majority of hospitals administered antibiotics to children following successful air enema reductions. In 2011, the World Health Organization (WHO) published a warning regarding the risk of injudicious antibiotic use [6]. As the root cause of antibiotic resistance, it has become a global public health problem. To fight drug resistance, the Ministry of Health in China conducted a remediation regarding the clinical use of antibiotics to curb their overuse, primarily at large hospitals [7]. Young children between 28 days and 3 years old require specific concerns; therefore, the rationale for antibiotic use in this population is an important research field. However, only a limited number of studies have been conducted regarding children suffering from intussusception. We therefore evaluated the use of antibiotics in young children with intussusception after successful air enema reduction.

## Materials and Methods

The Institutional Review Board of the Second Hospital of Shandong University approved this retrospective study. Notes were reviewed about children treated for intussusception at the Department of Pediatric Surgery in our hospital between January 1, 2011 and December 30, 2013. Diagnostic information was coded K56.1 (International Classification of Diseases, 10^th^ revision). Informed consent was signed by the parents of each child before the air enema was administered. Surgery was necessary when the air enema either failed or was incomplete [1]. The inclusion criteria included the first episode of intussusception and reduction with an air enema. The exclusion criteria included an age older than 3 years and surgery due to an unsuccessful air enema. Finally, 234 children with intussusception were recruited.

Phase I lasted from January 1, 2011 to October 31, 2011, during which our hospital did not utilize strict measures to normalize the use of antibiotics. We referred to this period as “preintervention.” Phase II lasted from November 1, 2011 to December 31, 2013, during which our hospital began to standardize doctors’ prescriptions, using a series of documents and incentives in response to the national policy. This phase was referred to as “postintervention.”

Demographic data, regimens, varieties, intentions and classifications were collected from the Center of Information, Department of Pediatric Surgery, Medical Records and Pharmacy (S1 Table). Antibiotics were classified as penicillins, cephalosporins, macrolides, imidazole derivatives, and other β-lactams.

The variables were described as follows: n, median, min, max and proportion. The predominance of each variable between the two phases was compared using χ^2^ test for categorical variables. All analyses were performed using SAS, version 9.4 (SAS Institute, Cary, NC, USA), and a *P* value less than 0.05 was statistically significant.

## Results

### Comparison of antibiotic use and intention in 234 children with intussusception in two phases

Of the 234 children in our study, 143 were boys (61%) and 91 were girls (39%). The overall median age was 16 months (range, 2–36). 55% (129/234) of the children had mesenteric lymphoid hyperplasia and lymphadenitis according to the imageological diagnosis. 68 and 166 children were included in two phases, respectively.

In phase I, the proportion of antibiotics usage was higher; 67 children received antibiotics following reduction, accounting for 99% (67/68), whereas 1% (1/68) did not. 84% of 67 children were prescribed antibiotics following air enema reduction for the purpose of preventing infection (56/67). In phase II, the proportion of antibiotic use decreased to 18% (30/166). 87% of antibiotics were used for treatment (26/30), and only 13% for prevention (4/30).

The overall antibiotic use rate after successful air enema reduction was 41% (97/234). The proportion and intention of antibiotic use had improved significantly after policy intervention (*P*<0.01). Single or two antibiotics in two phases had no differences (*P*>0.05). The results are included in Table 1.

**Table 1:** Antibiotic comparisons in children with intussusception after air enema reduction in two phases (n,%).

	Phase I	Phase II	*P*
**Total cases (234 children)**	68	166	
No antibiotic use	1 (1%)	136 (82%)	0.000
Antibiotic use	67 (99%)	30 (18%)	
**Intention (97 children)**	67	30	
Prevention	56 (84%)	4 (13%)	0.000
Treatment	11 (16%)	26 (87%)	
**Variety (97 children)**	67	30	
Single antibiotic	63 (94%)	27 (90%)	0.478
Two antibiotics	4 (6%)	3 (10%)	

Phase I: preintervention; Phase II: postintervention

### Comparison of prescription quantities of the antibiotics in two phases

After analyzing the antibiotics applied to 97 children, twelve types of antibiotics were used. Only cefaclor for suspension was administered orally; the others were given parenterally. The antibiotics included penicillins, 1^st^ generation cephalosporins, 2^nd^ generation cephalosporins, 3^rd^ generation cephalosporins, other β-lactams, macrolides and imidazole derivatives. In phase I, children with intussusception used a total of six types of antimicrobial agents, whereas ten types were used in phase II (Table 2). Aztreonam and cefamandole were the most frequently used antibacterials in phase I. They were used less frequently in phase II.

**Table 2:** Prescription quantity of antibiotics used to 97 children with intussusception (n,%).

Classification	Antibiotic name	Phase I	Phase II
penicillin	Flucloxacillin Sodium for Injection	/	1 (3%)
1st generation cephalosporin	Cefathiamidine for Injection	1 (1%)	1 (3%)
	Cefazolin Sodium Pentahydrate for Injection	/	5 (15%)
2nd generation cephalosporin	Cefamandole Nafate for Injection	18 (25%)	1 (3%)
	Cefotiam Hydrochloride for Injection	2 (3%)	6 (18%)
	Cefaclor for Suspension	/	3 (9%)
3rd generation cephalosporin	Ceftizoxime Sodium for Injection	/	8 (24%)
other β-lactams	Aztreonam for Injection	45 (63%)	4 (12%)
	Latamoxef Sodium for Injection	2 (3%)	/
	Cefoxitin Sodium for Injection	/	3 (9%)
macrolides	Azithromycin Lactobionate for Injection	/	1 (3%)
imidazole derivatives	Ornidazole and Sodium Chloride Injection	3 (4%)	/
Total		71 (100%)	33(100%)

Phase I: preintervention; Phase II: postintervention

## Discussion

Intussusception is one of the leading causes of intestinal obstruction among pediatric patients, with a male:female ratio of approximately 3:2 or 2:1[1]. Abdominal pain is the most common presenting complaint. Ileocolic intussusception represents 90% of all cases of intussusception, which is often multifactorial, as its underlying pathophysiology is largely unknown. The importance of early diagnosis and prompt non-operative intervention are essential in ensuring a good outcome [8].Complications such as fever, diarrhea, bloody stool, ischemia colitis, intestinal perforation and intestinal necrosis may occur, and may result in bowel resection and death if left untreated [9]. Therefore, in China’s hospitals, pediatricians generally believe that the administration of appropriate antibiotics and nutritional rehydration are necessary following air enema reduction to properly address energy consumption, endotoxin absorption, intestinal disorders and infectious factors. However, it must be acknowledged that abuse exists regarding the antibiotics use among children with intussusception. Since 2011, China has utilized the Administrative Measures for the Clinical Use of Antibacterial Drugs [7]. In response to it, our hospital organized one experts team, formulated antibiotic management guidelines, issued directories and prescriptions of antibacterial drug.

From our study, we found antibiotics abuse existed widespread before policy intervention, because most of children had no indications of antimicrobial application. In phase I, almost 99% of the enrolled children received antibiotics, and as many as 84% received medication as prophylaxis. For example of aztreonam, in Phase I, among forty-five children with it, only five children had fever and leukocytosis. And in Phase II, only one child had fever among four children prescribed with it. Other forty-three children did not have any other abnormalities. In other words, 88% of children actually had no medication indications. Physicians used antimicrobial agents mainly considering that air emema may cause pediatric intestinal mucosal hyperemia or damage. Antibiotic use may prevent infection. Moreover, physicians' desicions were interferred by parents' strong appeals.

The other abuse was the inadequate usage of antibiotics. In this study, aztreonam was used for one children with respiratory tract infection, two with mesenteric lymphadenitis, four with mesenteric lymphoid hyperplasia. Above regimens were inappropriate. Aztreonam should be used only for aerobic gram-negative bacterial infections due to its narrow antimicrobial spectrum. Excessive use may cause pathogenic bacteria to develop resistance; therefore, it must be strictly controlled. In 1987, aztreonam was approved by the FDA, but it was restricted to children 12 years and older [10]. Although latamoxef (3%) had previously exhibited good antibacterial activity against Enterobacteriaceae, it also caused decreased prothrombin levels, thrombocytopenia and bleeding [11]. Therefore, it was not recommended to children with intussusception. Ornidazole (4%), which were against anaerobic bacteria, was not recommended for children under 3 years old according to the Chinese National Formulary (Children’s Edition) [8].

The discovery of antibiotics in the 1930s fundamentally transformed the way physicians care for patients, shifting their focus from diagnoses to a treatment-based approach that saved lives[12]. However, the increased use of antibiotics in the community eliminates weaker bacteria and selects for the stronger organisms, which results in bacterial resistance to multiple antibiotics [6]. Routine antibiotic administration before enema reduction of intussusception is advocated in the book of USA Pediatric Surgery, which states that broad-spectrum antibiotics should be administered during any situation in which the vascular supply to the bowel may be jeopardized [1]. The Children’s Hospital of Illinois in USA recommends that before treating intussusception, one dose of cefoxitin (40 mg/kg) should be administered. Although perforation is rare, this provides antibiotic coverage against any potential infectious complications that may arise. Among patients with gross peritonitis, coverage with broad-spectrum antibiotics is necessary; the appropriate length of treatment should be determined once the degree of contamination has been determined[13]. However, one study in Canada determined that administering antibiotics before the enema reduction of intussusception was of limited value [4]. The Japanese Guidelines for the Management of Intussusception in Children (2011) also stated that the routine administration of antimicrobial drugs is not necessary following enema reduction. Serial blood cultures, both pre-enema and post-enema, do not justify the routine administration of antibiotics following enema reduction. The risk of bacteremia from enteric pathogens following air enema for the reduction of intussusception appears to be low [14]. Antimicrobial therapy is justified only in the presence of underlying sepsis.

Based on the results of our study, we recommend that pediatricians should continue to observe the children for at least 24 hours following the successful reduction of intussusception. To manage coexisting infectious diseases, including mesenteric lymphadenitis, upper respiratory tract infections, bronchitis, bronchopneumonia and enteritis, appropriate antimicrobial agents may be used. β-lactams and macrolides are the most appropriate antibiotics for these conditions.

Our study also had some limitations. If this study was a multicenter surveillance study, the results would be more convincing. Because of the secrecy surrounding the study, many large hospitals were reluctant to provide their medication data. In spite of this, we observed the influence of national policy on antibiotic use. We hope that our surveillance will provide evidence-based pharmacy for children with intussusception.

## Supporting Information

S1 Table Database of antibiotic use for young children with intussusception. (XLS)
